# Technology to Support Self-Regulation in Older Adulthood: Insights From Design to Implementation

**DOI:** 10.1093/geroni/igab046.475

**Published:** 2021-12-17

**Authors:** Shannon Meija, Hans-Werner Wahl

## Abstract

Across the lifespan, individuals adapt to change through the careful monitoring and adjustment of goals, demands, and performance—processes of self-regulation. Technology in support of self-regulatory processes may compensate for deficiencies in the ability to set, monitor, and work toward goals. Our purpose in this symposium is to forward the discourse on how health technology—from design to implementation—can assist older adults in their efforts to support their health and well-being in daily life. Our symposium begins with design considerations for technologies that support processes of information seeking, reflection, and action. Chin presents a process for designing conversation agents that guide dialogues with older adults to support informal self-regulated learning of health information. Nie and colleagues synthesize the literature on visual feedback to provide a framework that illustrates how visual design elements can link feedback to action. The symposium continues with papers that speak to older adults’ experiences using technology to accomplish their goals. Mejía and colleagues use insight from older adults who had self-assessed their balance for 30 consecutive days to explore themes of self-monitoring accuracy and feedback preferences. Francis and colleagues use data from the Detroit-based Social Relations Study to illustrate how technology use and its implications vary when older adults engage with their weaker social ties. The symposium will conclude with a discussion led by Wahl, who will situate the papers, and the discourse on health technology design and application, within lifespan developmental and action perspectives on aging.

